# Supplementary material for: Canine osteosarcoma genome sequencing identifies recurrent mutations in DMD and the histone methyltransferase gene SETD2
Source: Commun Biol. 2019 Jul 19;2:266. doi: 10.1038/s42003-019-0487-2 (PMC6642146; doi:10.1038/s42003-019-0487-2)
Supplement: Supplementary file 1 — Supplementary Information [file 42003_2019_487_MOESM1_ESM.pdf]

Supplemental Figure 1

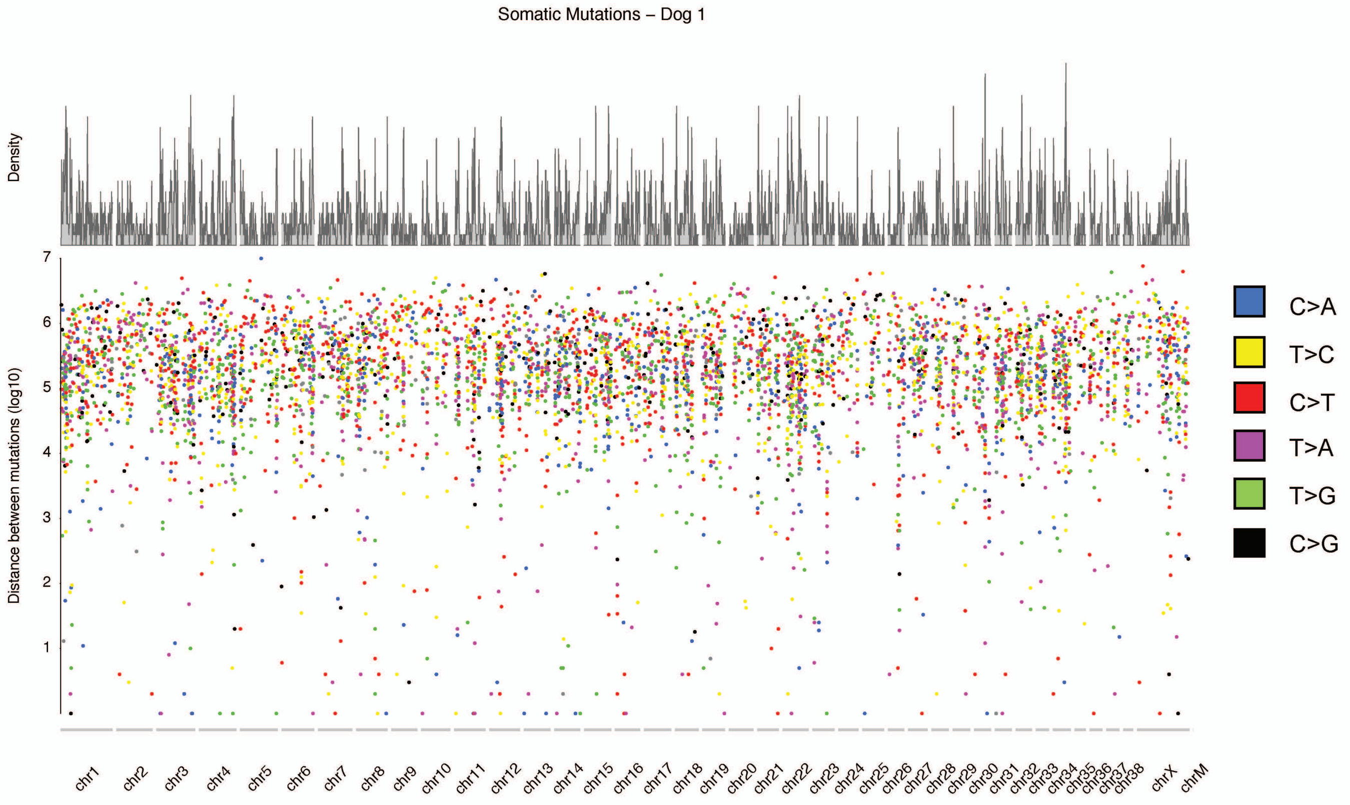

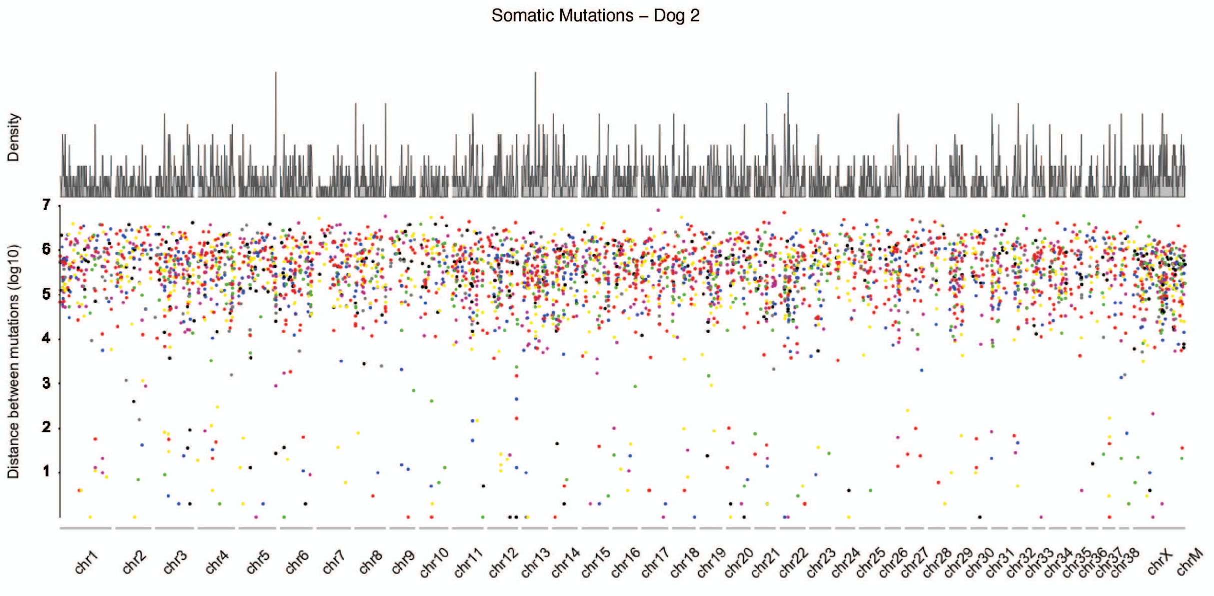

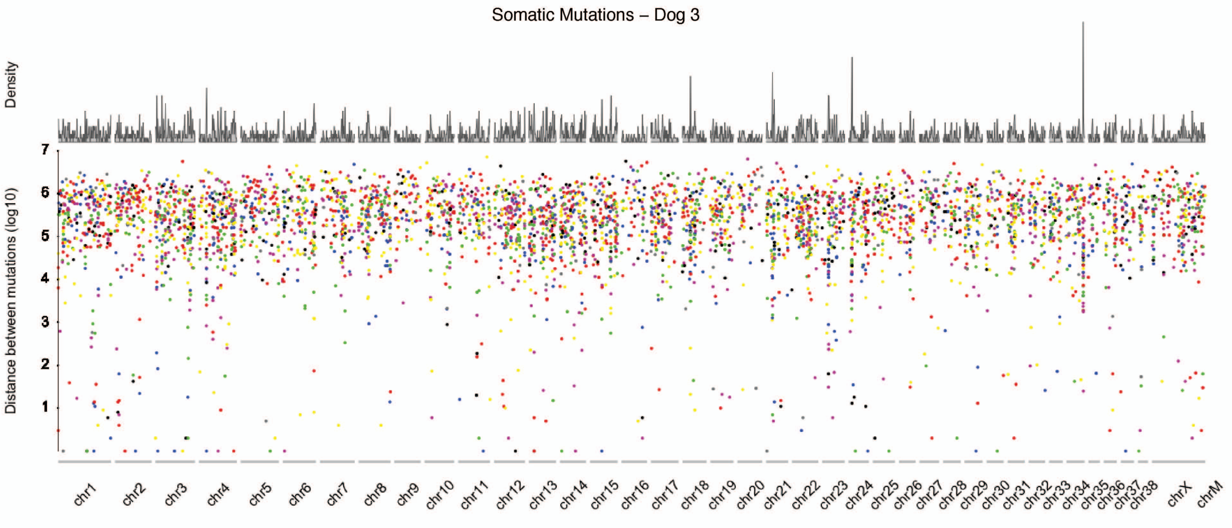

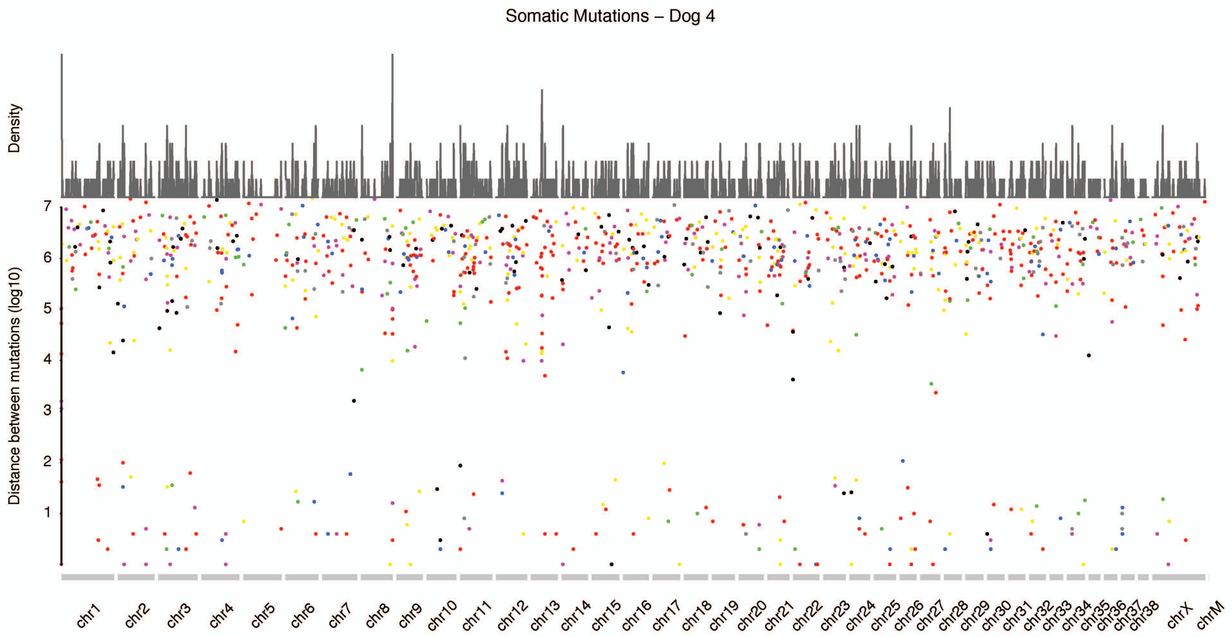
Somatic Mutations – Dog 4
Density
Distance between mutations (log10)
7
6
5
4
3
2
1
chr1 chr2 chr3 chr4 chr5 chr6 chr7 chr8 chr9 chr10 chr11 chr12 chr13 chr14 chr15 chr16 chr17 chr18 chr19 chr20 chr21 chr22 chr23 chr24 chr25 chr26 chr27 chr28 chr29 chr30 chr31 chr32 chr33 chr34 chr35 chr36 chr37 chr38 chrX chrM

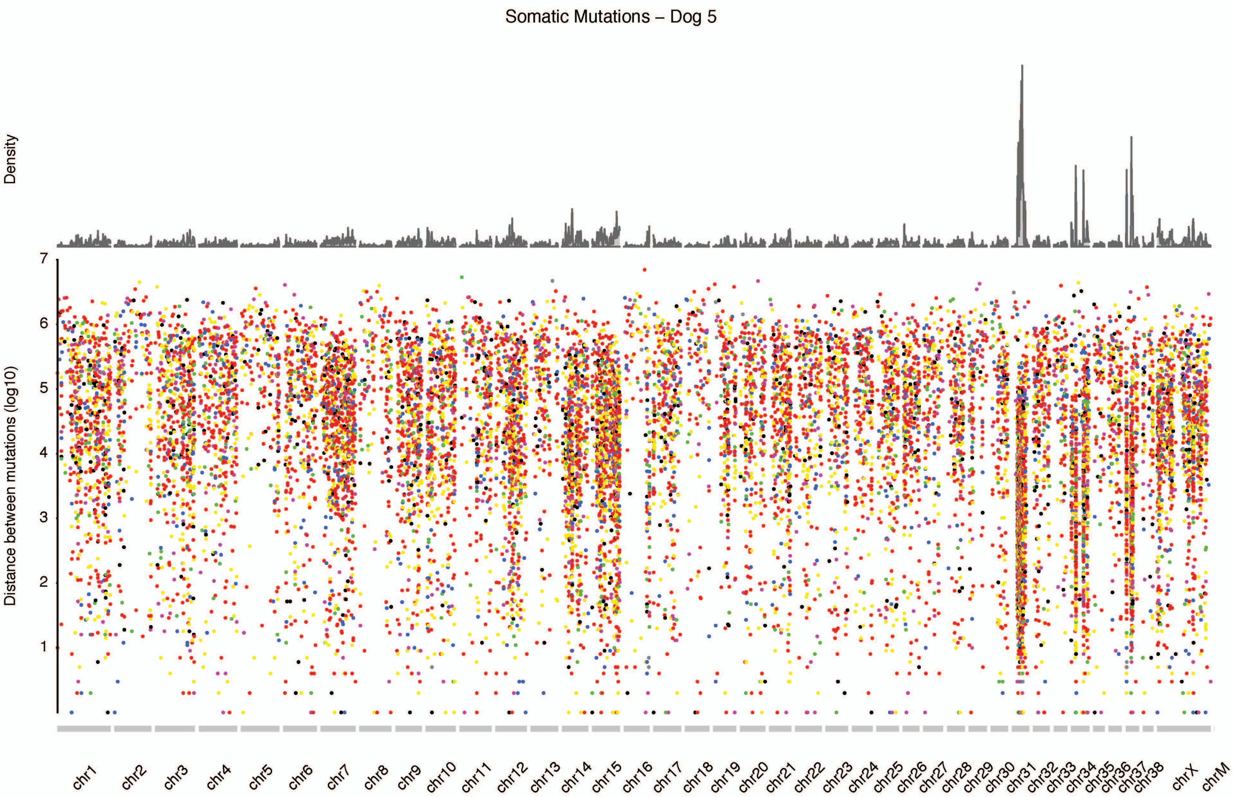
Somatic Mutations – Dog 5
Density
Distance between mutations (log10)
7
6
5
4
3
2
1
chr1 chr2 chr3 chr4 chr5 chr6 chr7 chr8 chr9 chr10 chr11 chr12 chr13 chr14 chr15 chr16 chr17 chr18 chr19 chr20 chr21 chr22 chr23 chr24 chr25 chr26 chr27 chr28 chr29 chr30 chr31 chr32 chr33 chr34 chr35 chr36 chr37 chr38 chrX chrM

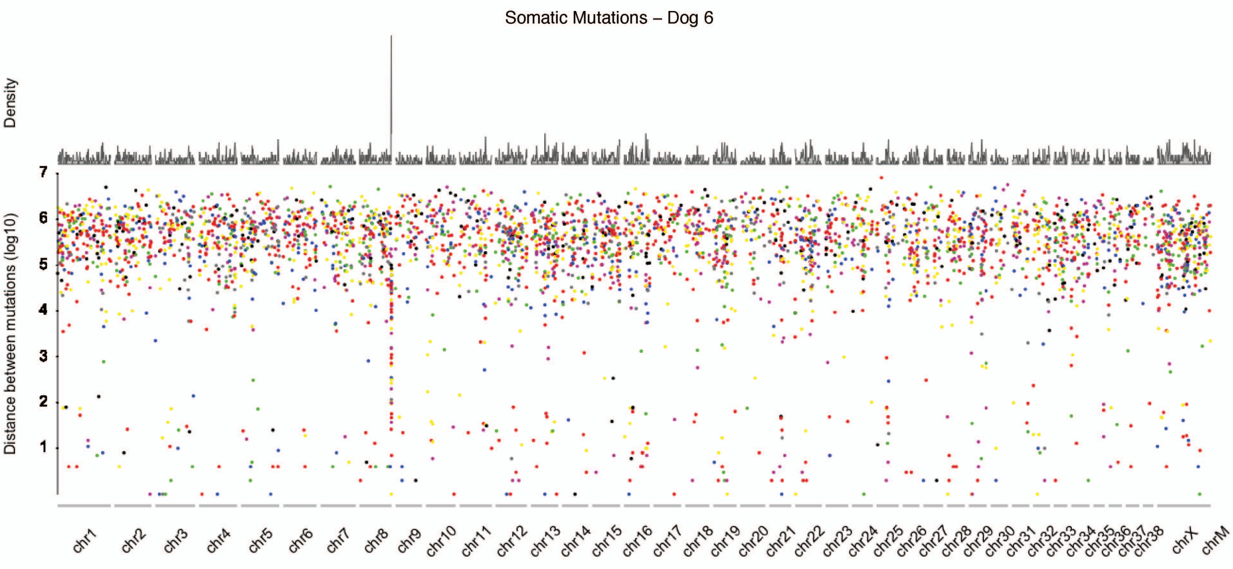
Somatic Mutations – Dog 6
Density
Distance between mutations (log10)
7
6
5
4
3
2
1
chr1 chr2 chr3 chr4 chr5 chr6 chr7 chr8 chr9 chr10 chr11 chr12 chr13 chr14 chr15 chr16 chr17 chr18 chr19 chr20 chr21 chr22 chr23 chr24 chr25 chr26 chr27 chr28 chr29 chr30 chr31 chr32 chr33 chr34 chr35 chr36 chr37 chr38 chrX chrM

Somatic Mutations – Dog 7

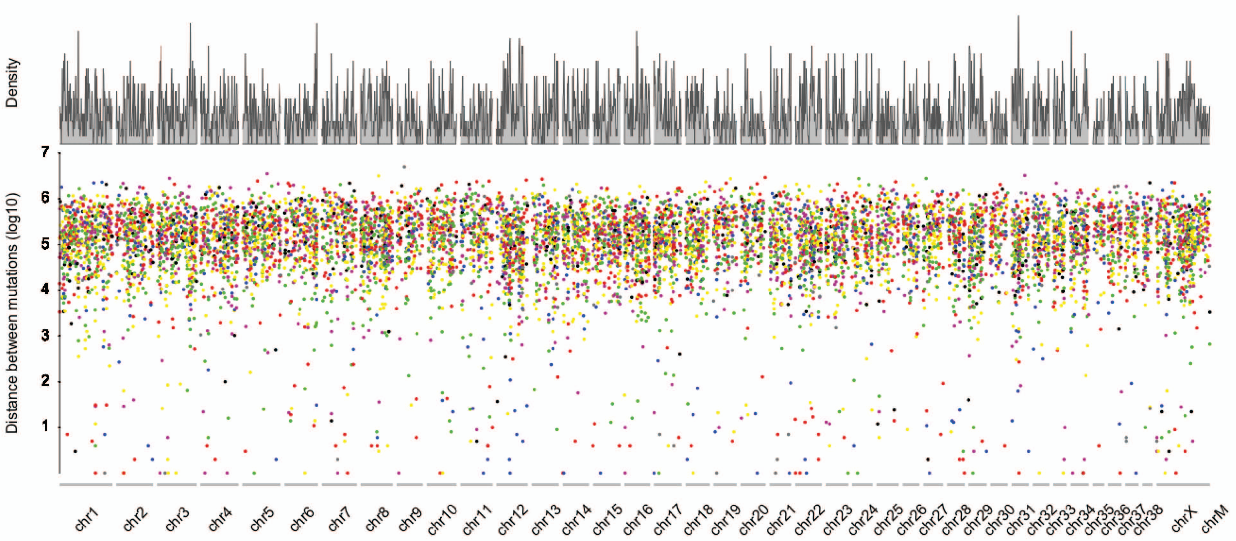

Somatic Mutations – Dog 8

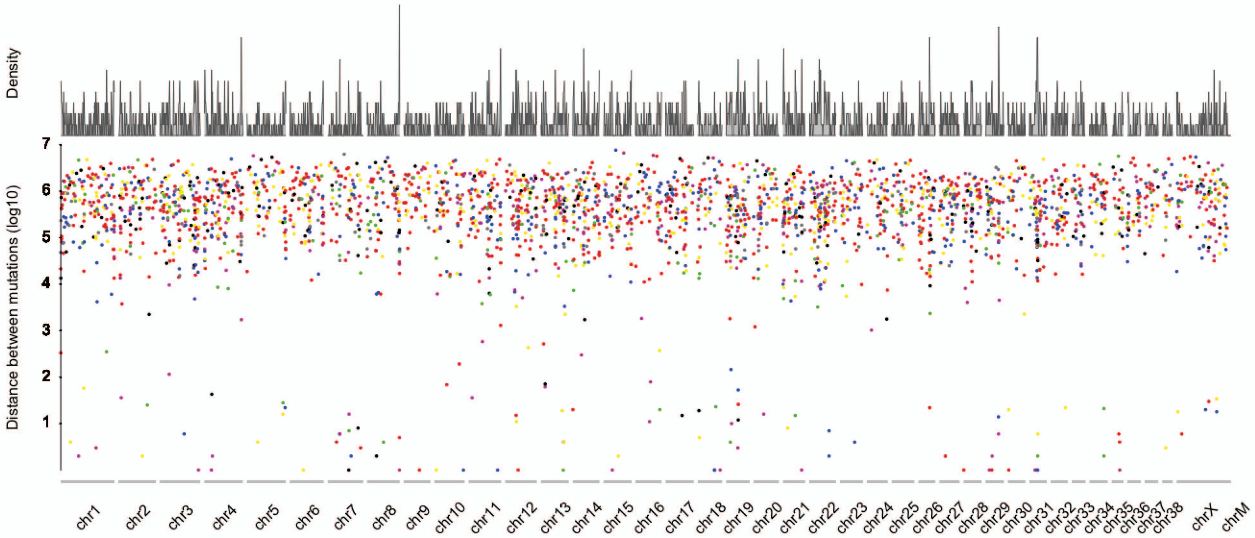

Somatic Mutations – Dog 9

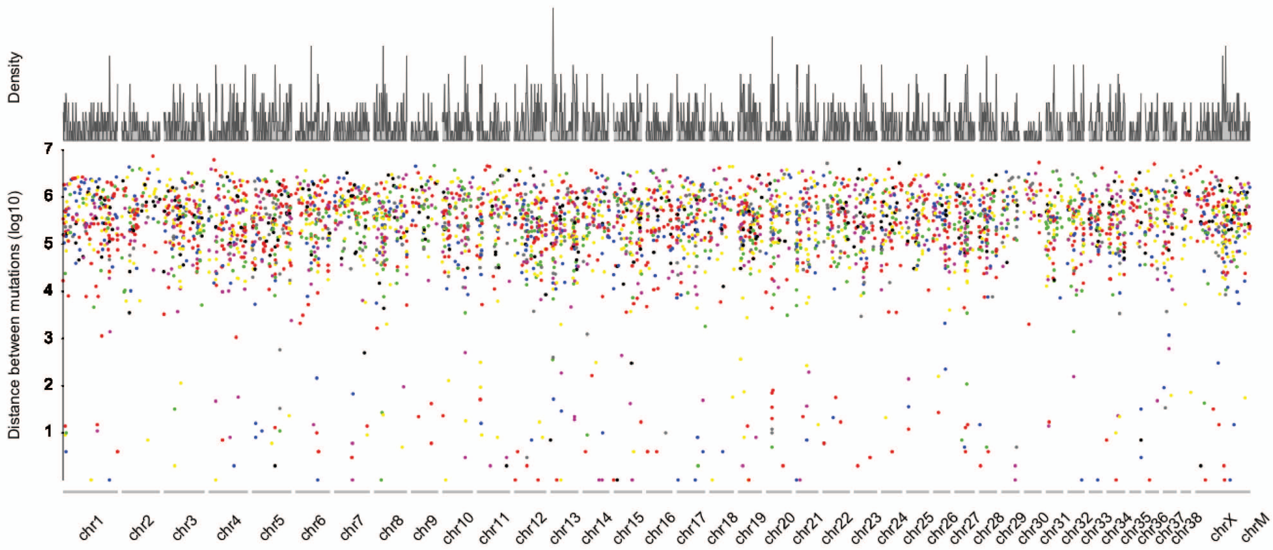

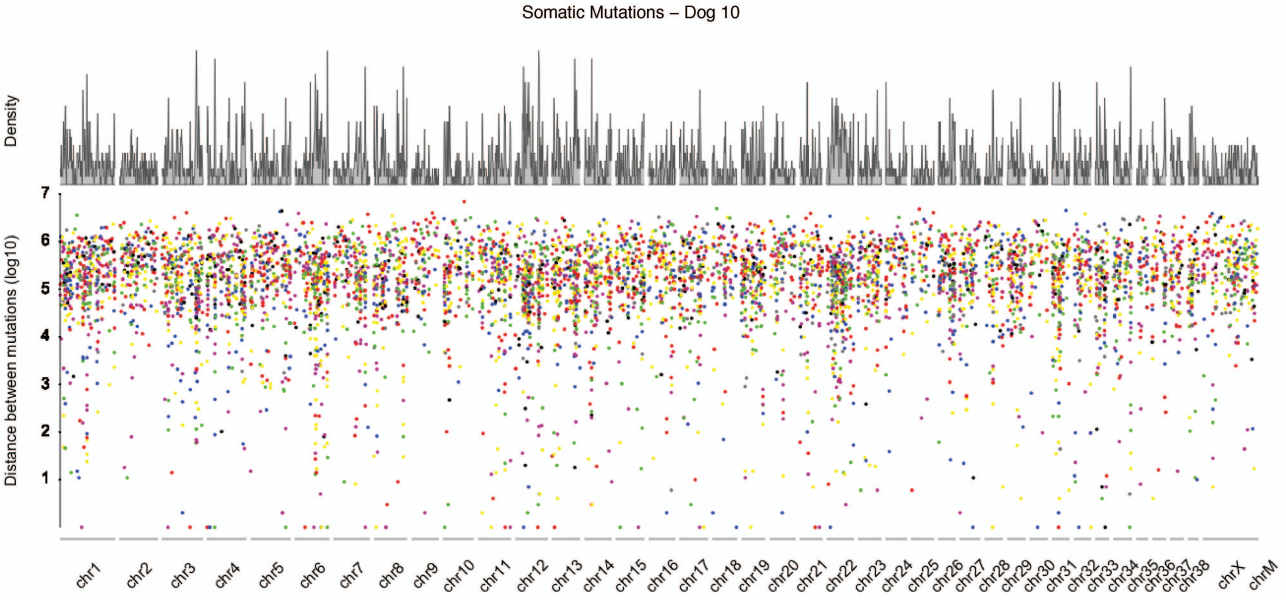
Somatic Mutations – Dog 10
Density
Distance between mutations (log10)
1 2 3 4 5 6 7
chr1 chr2 chr3 chr4 chr5 chr6 chr7 chr8 chr9 chr10 chr11 chr12 chr13 chr14 chr15 chr16 chr17 chr18 chr19 chr20 chr21 chr22 chr23 chr24 chr25 chr26 chr27 chr28 chr29 chr30 chr31 chr32 chr33 chr34 chr35 chr36 chr37 chr38 chrX chrM

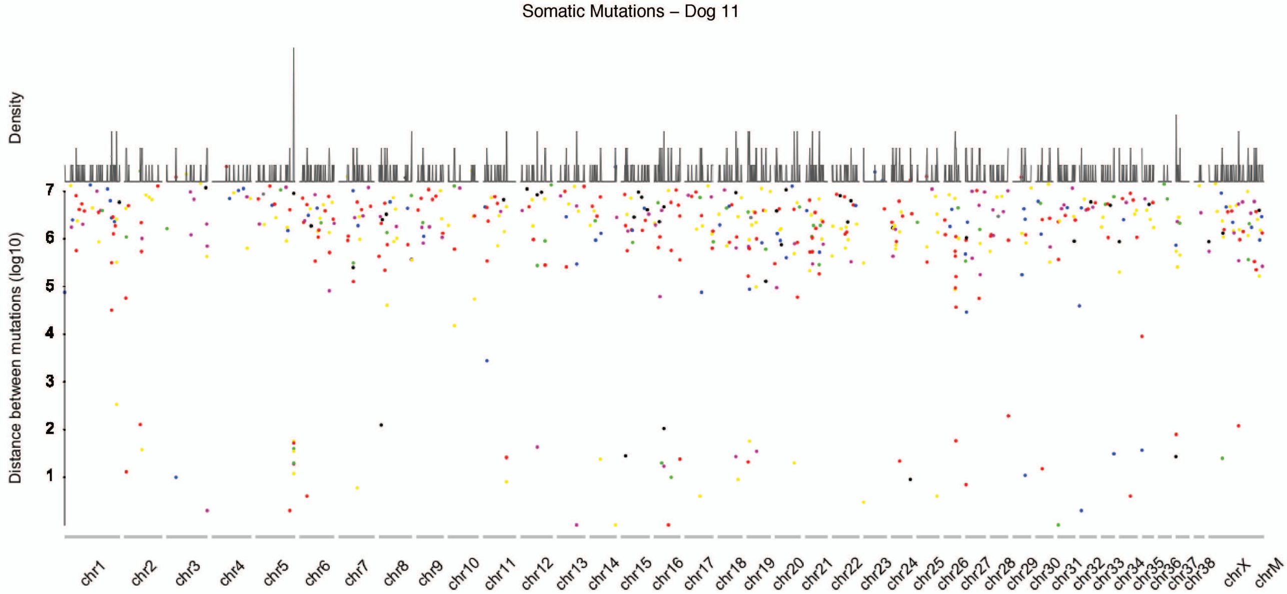
Somatic Mutations – Dog 11
Density
Distance between mutations (log10)
1 2 3 4 5 6 7
chr1 chr2 chr3 chr4 chr5 chr6 chr7 chr8 chr9 chr10 chr11 chr12 chr13 chr14 chr15 chr16 chr17 chr18 chr19 chr20 chr21 chr22 chr23 chr24 chr25 chr26 chr27 chr28 chr29 chr30 chr31 chr32 chr33 chr34 chr35 chr36 chr37 chr38 chrX chrM

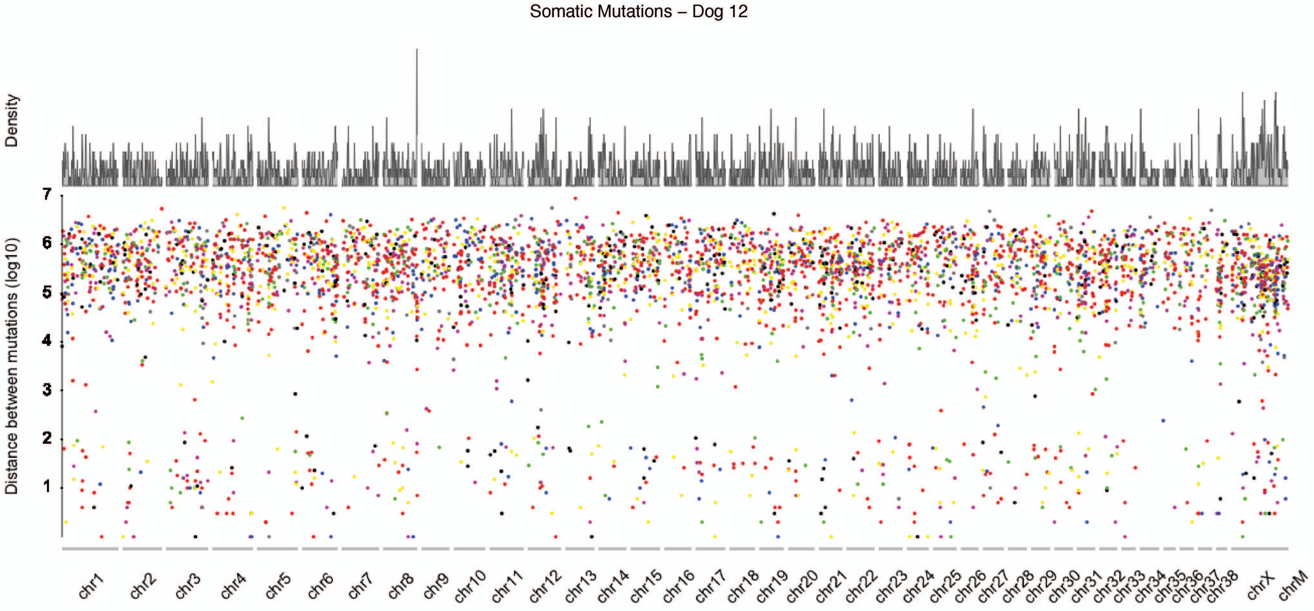
Somatic Mutations – Dog 12
Density
Distance between mutations (log10)
1 2 3 4 5 6 7
chr1 chr2 chr3 chr4 chr5 chr6 chr7 chr8 chr9 chr10 chr11 chr12 chr13 chr14 chr15 chr16 chr17 chr18 chr19 chr20 chr21 chr22 chr23 chr24 chr25 chr26 chr27 chr28 chr29 chr30 chr31 chr32 chr33 chr34 chr35 chr36 chr37 chr38 chrX chrM

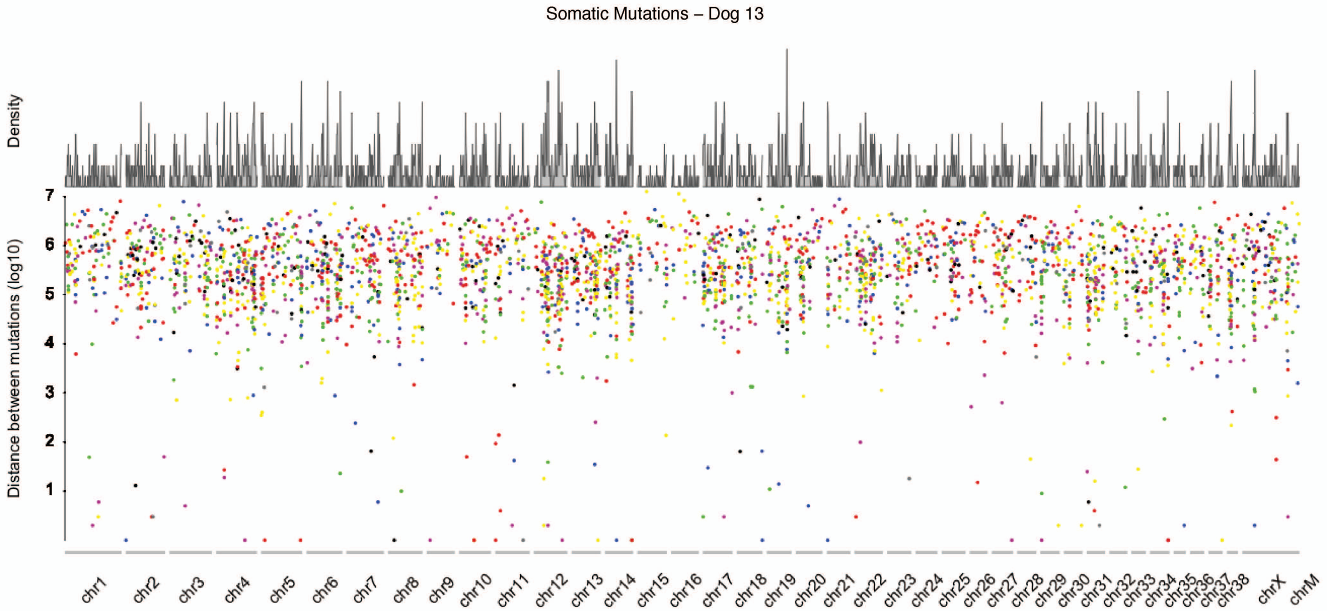
Somatic Mutations – Dog 13
Density
Distance between mutations (log10)
chr1 chr2 chr3 chr4 chr5 chr6 chr7 chr8 chr9 chr10 chr11 chr12 chr13 chr14 chr15 chr16 chr17 chr18 chr19 chr20 chr21 chr22 chr23 chr24 chr25 chr26 chr27 chr28 chr29 chr30 chr31 chr32 chr33 chr34 chr35 chr36 chr37 chr38 chrX chrM

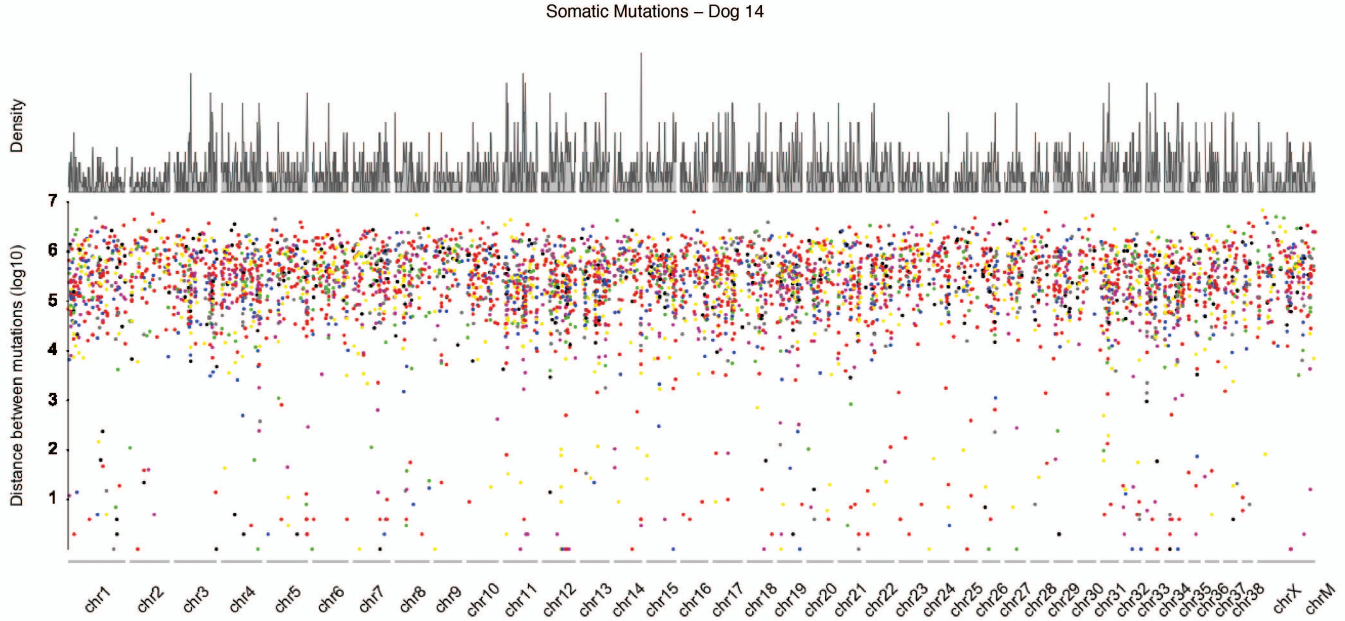
Somatic Mutations – Dog 14
Density
Distance between mutations (log10)
chr1 chr2 chr3 chr4 chr5 chr6 chr7 chr8 chr9 chr10 chr11 chr12 chr13 chr14 chr15 chr16 chr17 chr18 chr19 chr20 chr21 chr22 chr23 chr24 chr25 chr26 chr27 chr28 chr29 chr30 chr31 chr32 chr33 chr34 chr35 chr36 chr37 chr38 chrX chrM

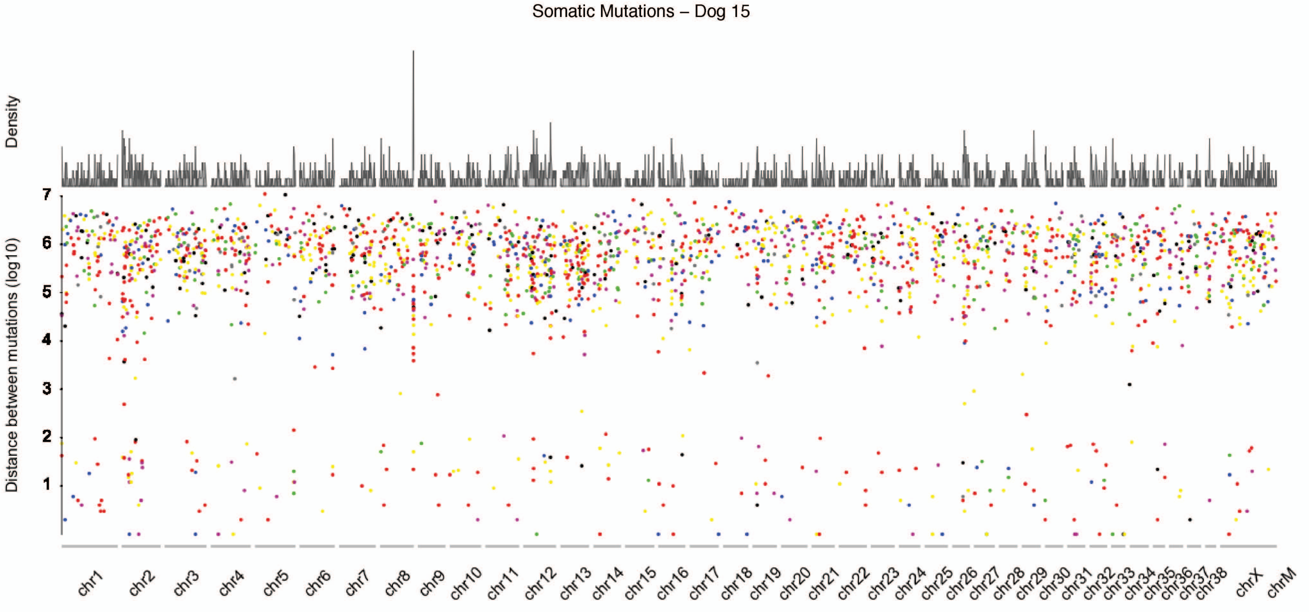
Somatic Mutations – Dog 15
Density
Distance between mutations (log10)
chr1 chr2 chr3 chr4 chr5 chr6 chr7 chr8 chr9 chr10 chr11 chr12 chr13 chr14 chr15 chr16 chr17 chr18 chr19 chr20 chr21 chr22 chr23 chr24 chr25 chr26 chr27 chr28 chr29 chr30 chr31 chr32 chr33 chr34 chr35 chr36 chr37 chr38 chrX chrM

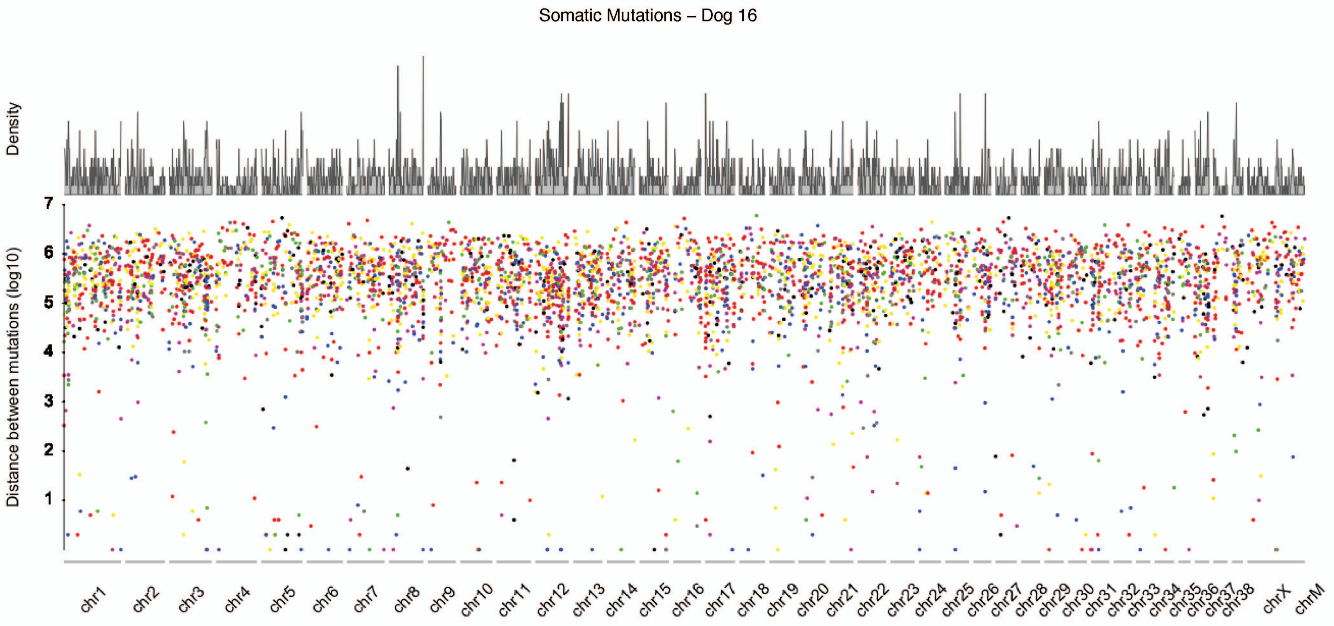
Somatic Mutations – Dog 16
Density
Distance between mutations (log10)
1 2 3 4 5 6 7
chr1 chr2 chr3 chr4 chr5 chr6 chr7 chr8 chr9 chr10 chr11 chr12 chr13 chr14 chr15 chr16 chr17 chr18 chr19 chr20 chr21 chr22 chr23 chr24 chr25 chr26 chr27 chr28 chr29 chr30 chr31 chr32 chr33 chr34 chr35 chr36 chr37 chr38 chrX chrM

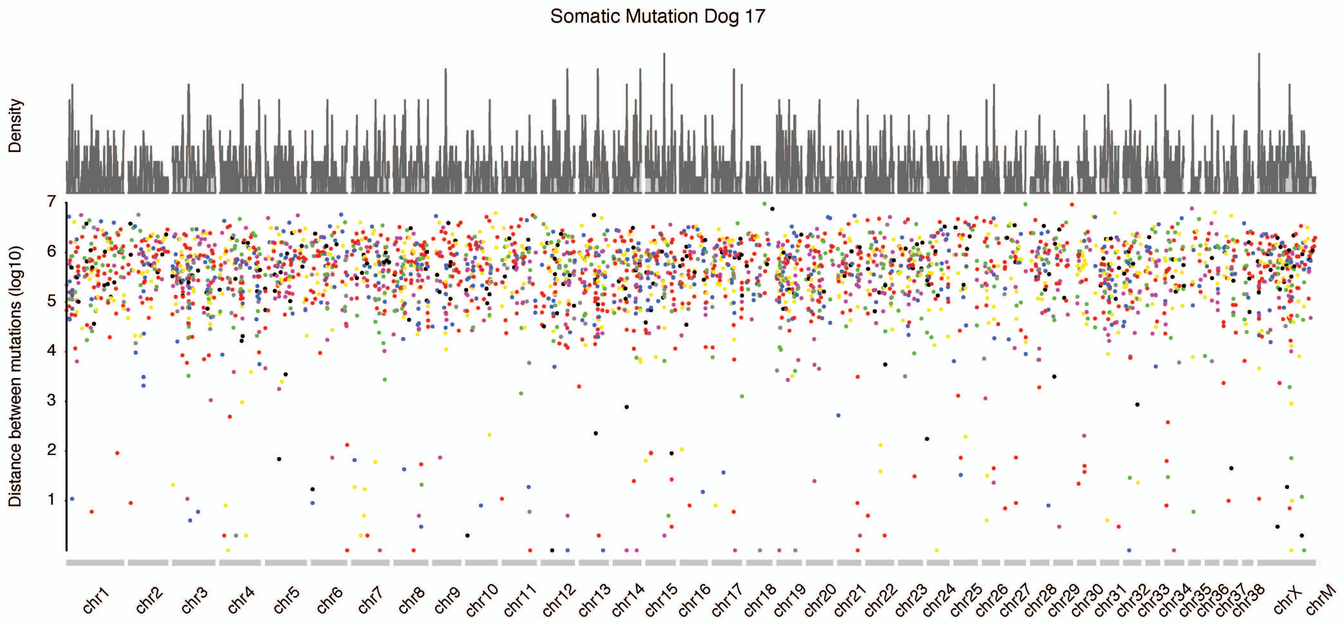
Somatic Mutation Dog 17
Density
Distance between mutations (log10)
1 2 3 4 5 6 7
chr1 chr2 chr3 chr4 chr5 chr6 chr7 chr8 chr9 chr10 chr11 chr12 chr13 chr14 chr15 chr16 chr17 chr18 chr19 chr20 chr21 chr22 chr23 chr24 chr25 chr26 chr27 chr28 chr29 chr30 chr31 chr32 chr33 chr34 chr35 chr36 chr37 chr38 chrX chrM

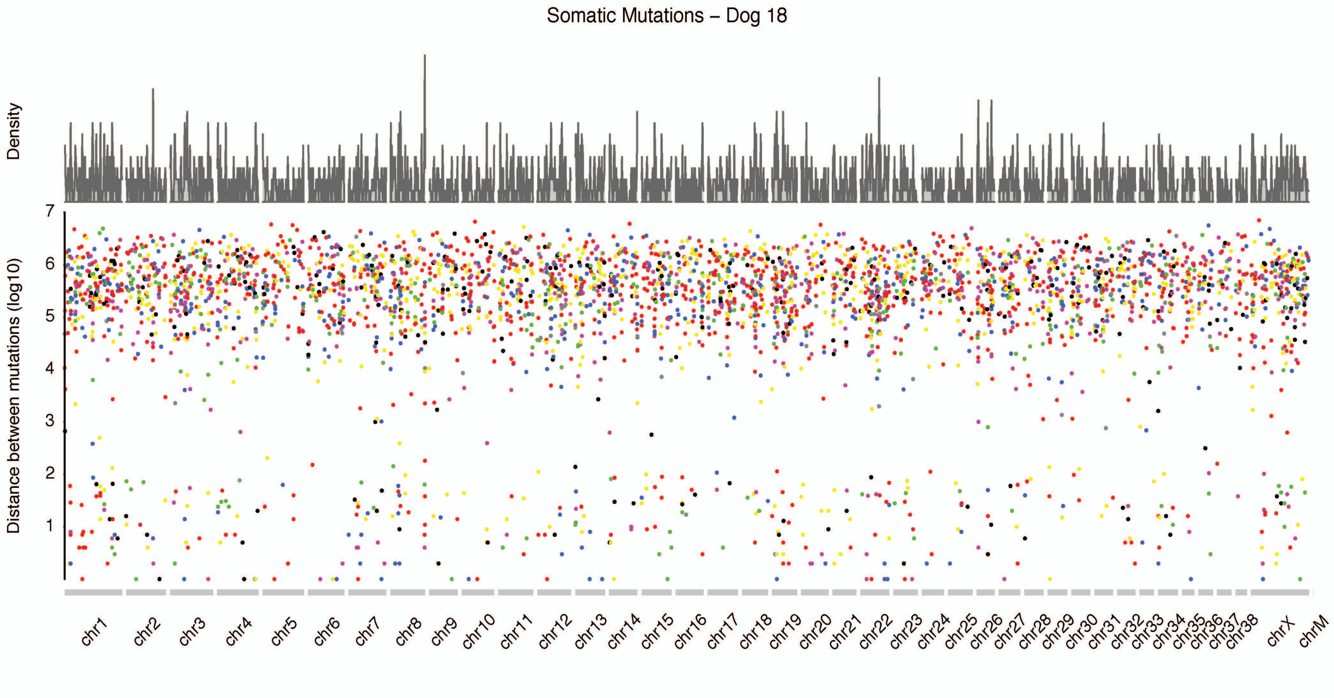
Somatic Mutations – Dog 18
Density
Distance between mutations (log10)
1 2 3 4 5 6 7
chr1 chr2 chr3 chr4 chr5 chr6 chr7 chr8 chr9 chr10 chr11 chr12 chr13 chr14 chr15 chr16 chr17 chr18 chr19 chr20 chr21 chr22 chr23 chr24 chr25 chr26 chr27 chr28 chr29 chr30 chr31 chr32 chr33 chr34 chr35 chr36 chr37 chr38 chrX chrM

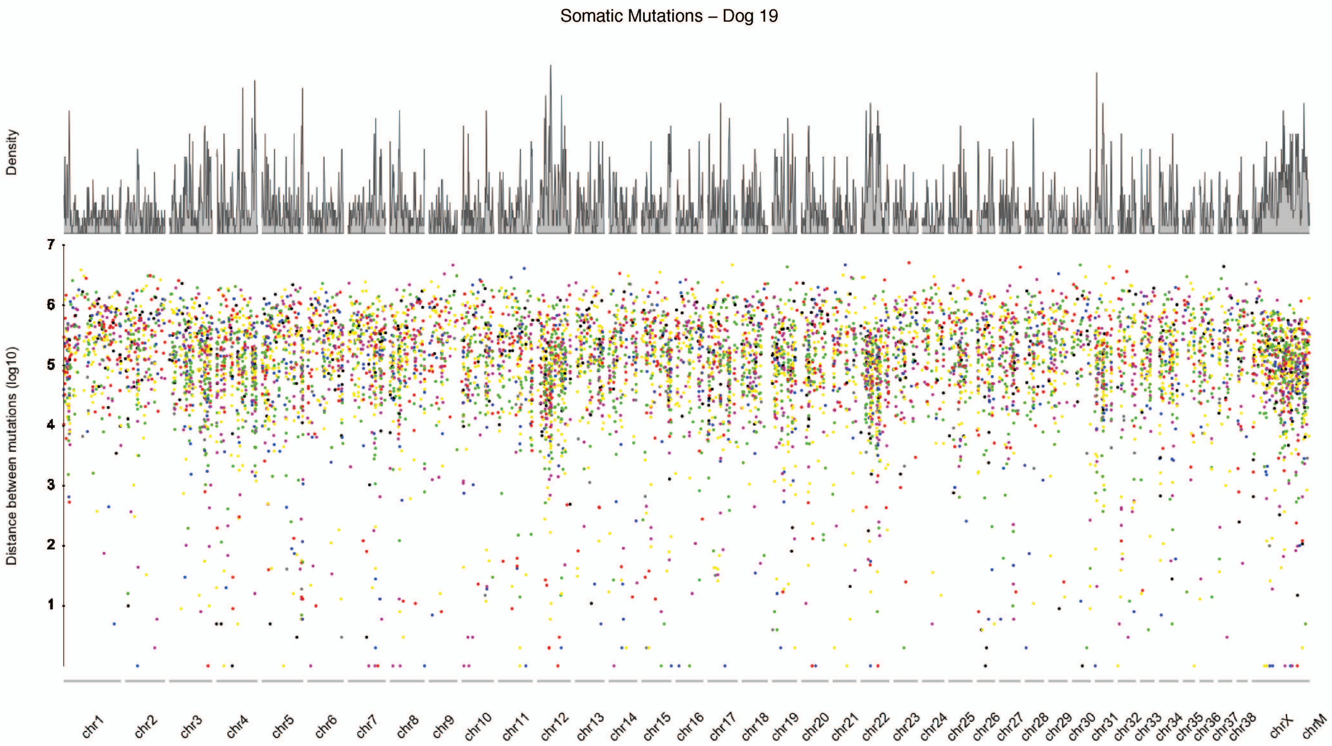
Somatic Mutations – Dog 19
Density
Distance between mutations (log10)
chr1 chr2 chr3 chr4 chr5 chr6 chr7 chr8 chr9 chr10 chr11 chr12 chr13 chr14 chr15 chr16 chr17 chr18 chr19 chr20 chr21 chr22 chr23 chr24 chr25 chr26 chr27 chr28 chr29 chr30 chr31 chr32 chr33 chr34 chr35 chr36 chr37 chr38 chrX chrM

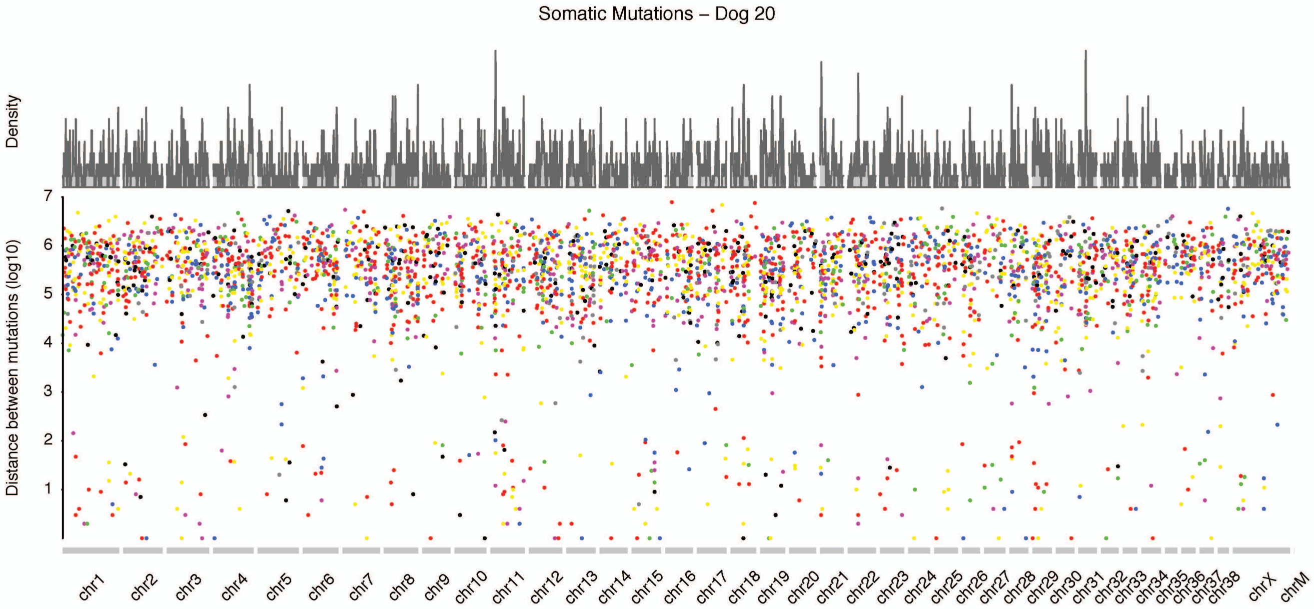
Somatic Mutations – Dog 20
Density
Distance between mutations (log10)
chr1 chr2 chr3 chr4 chr5 chr6 chr7 chr8 chr9 chr10 chr11 chr12 chr13 chr14 chr15 chr16 chr17 chr18 chr19 chr20 chr21 chr22 chr23 chr24 chr25 chr26 chr27 chr28 chr29 chr30 chr31 chr32 chr33 chr34 chr35 chr36 chr37 chr38 chrX chrM

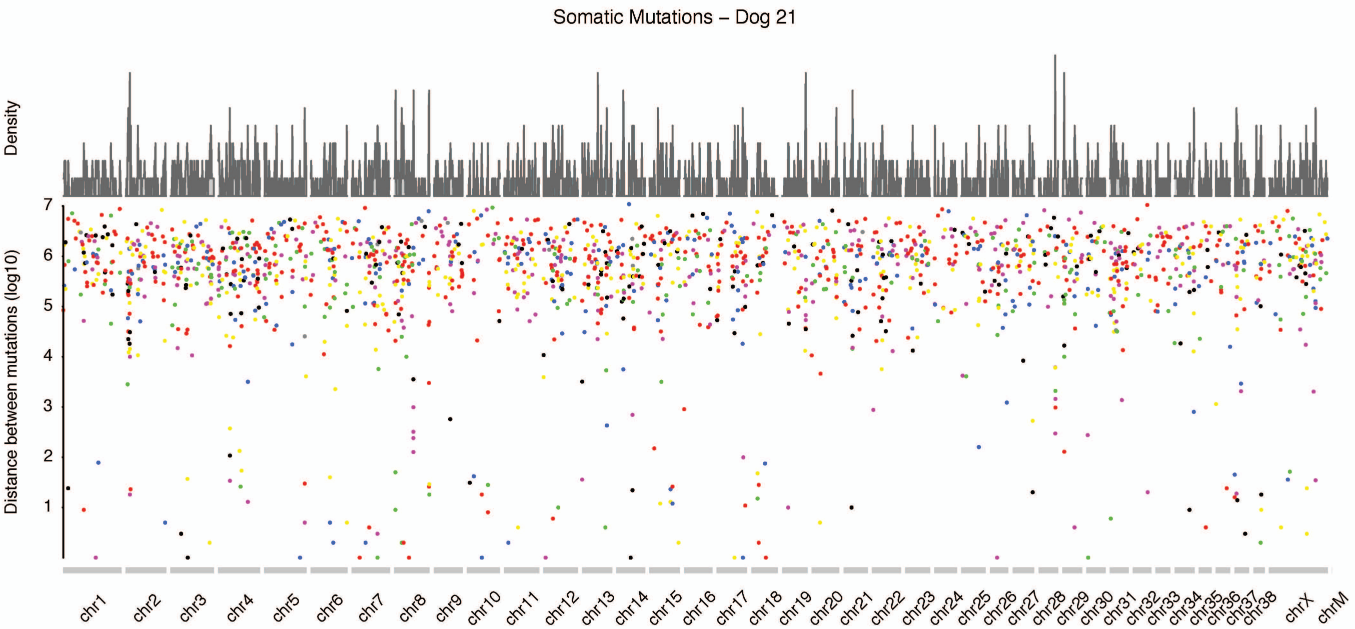
Somatic Mutations – Dog 21
Density
Distance between mutations (log10)
chr1 chr2 chr3 chr4 chr5 chr6 chr7 chr8 chr9 chr10 chr11 chr12 chr13 chr14 chr15 chr16 chr17 chr18 chr19 chr20 chr21 chr22 chr23 chr24 chr25 chr26 chr27 chr28 chr29 chr30 chr31 chr32 chr33 chr34 chr35 chr36 chr37 chr38 chrX chrM

Somatic Mutations – Dog 22

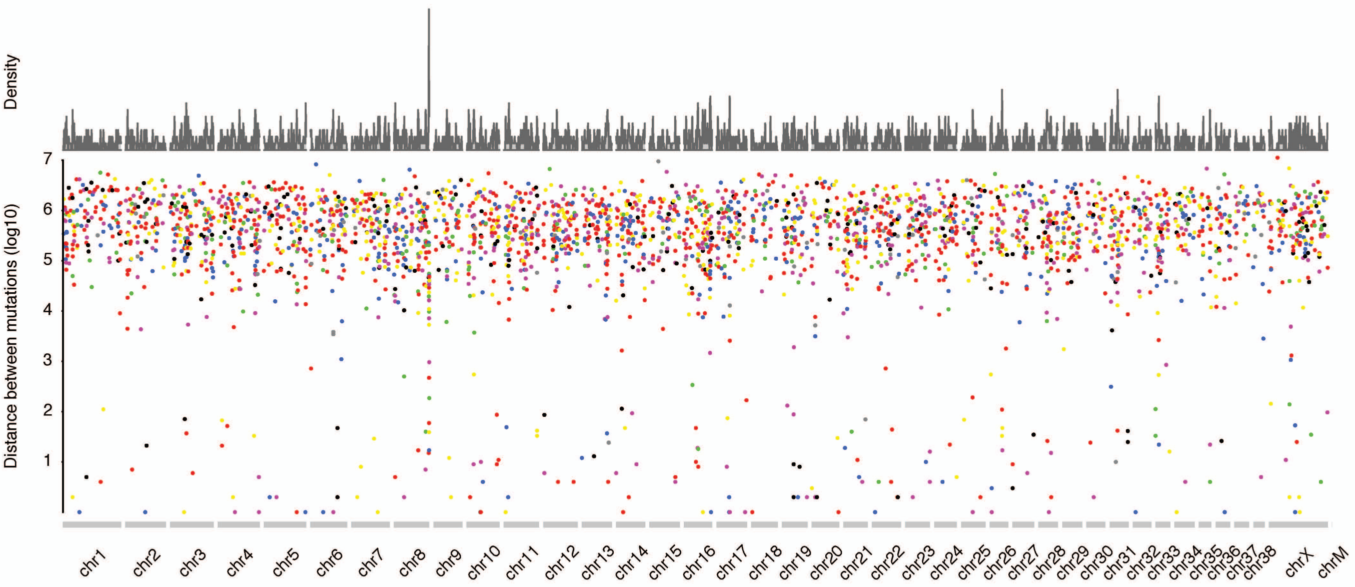

Somatic Mutations – Dog 23

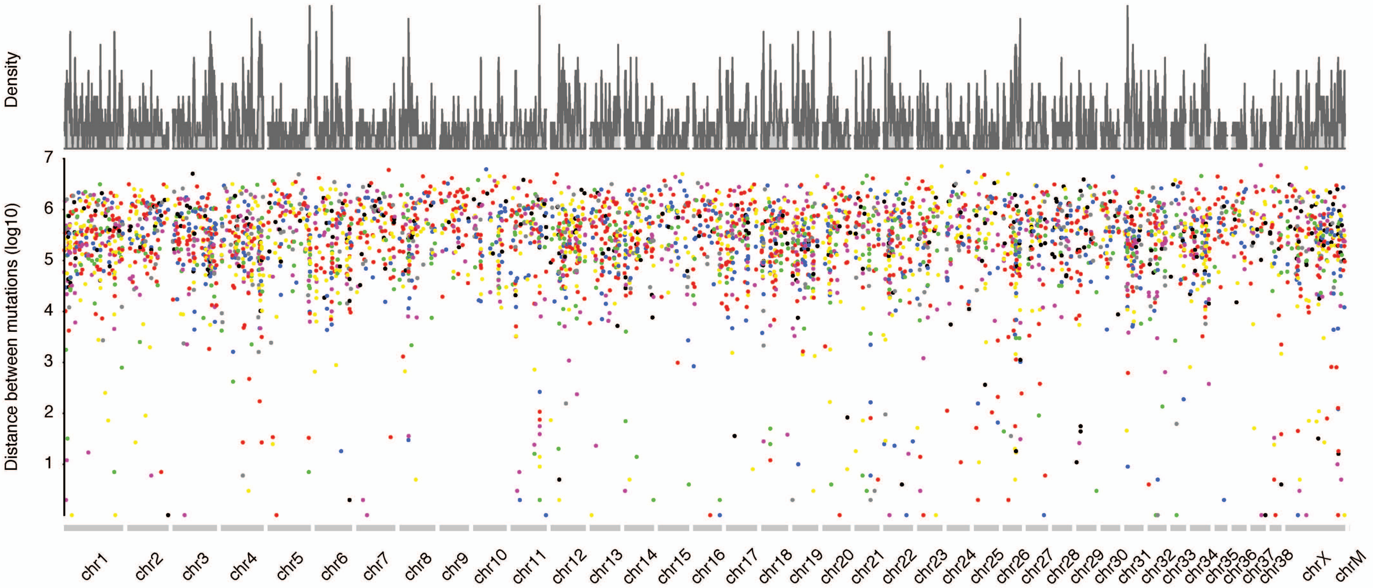

Somatic Mutations – Dog 24

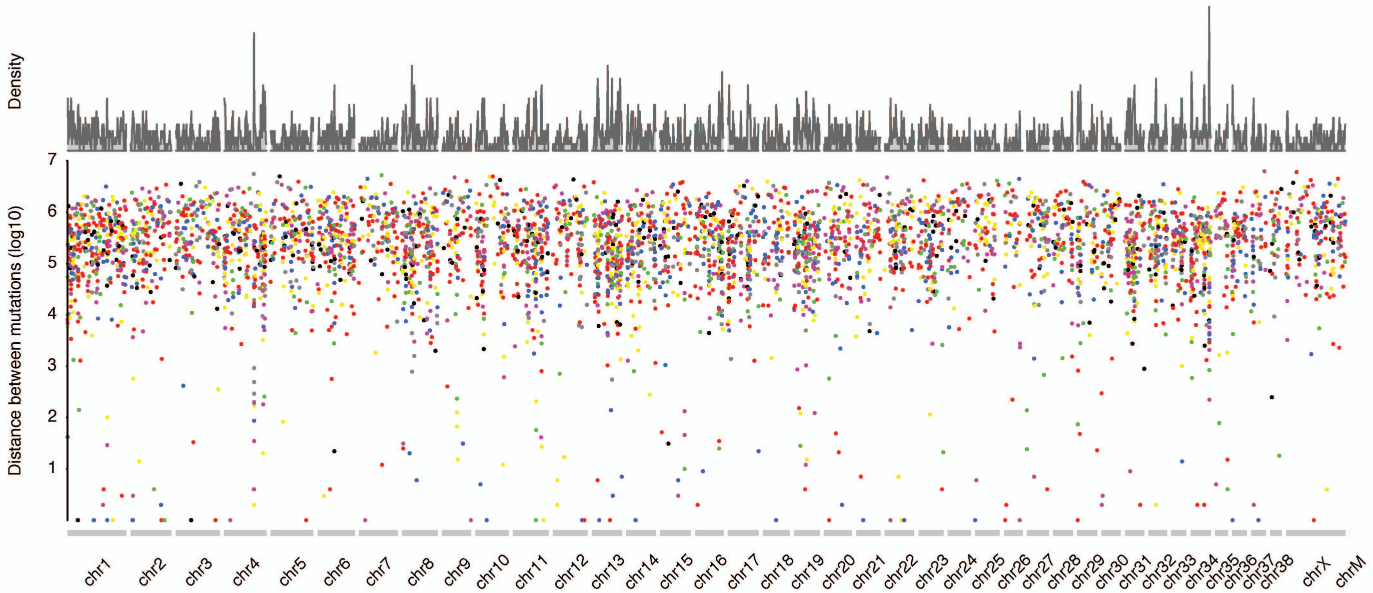

Supplemental Figure 1: Localized point mutations in WGS samples. Rainfall plots demonstrating the density and distribution of somatic mutations across all WGS samples. Basepair distance between events is represented on the y-axis.

Supplemental Figure 2

A.

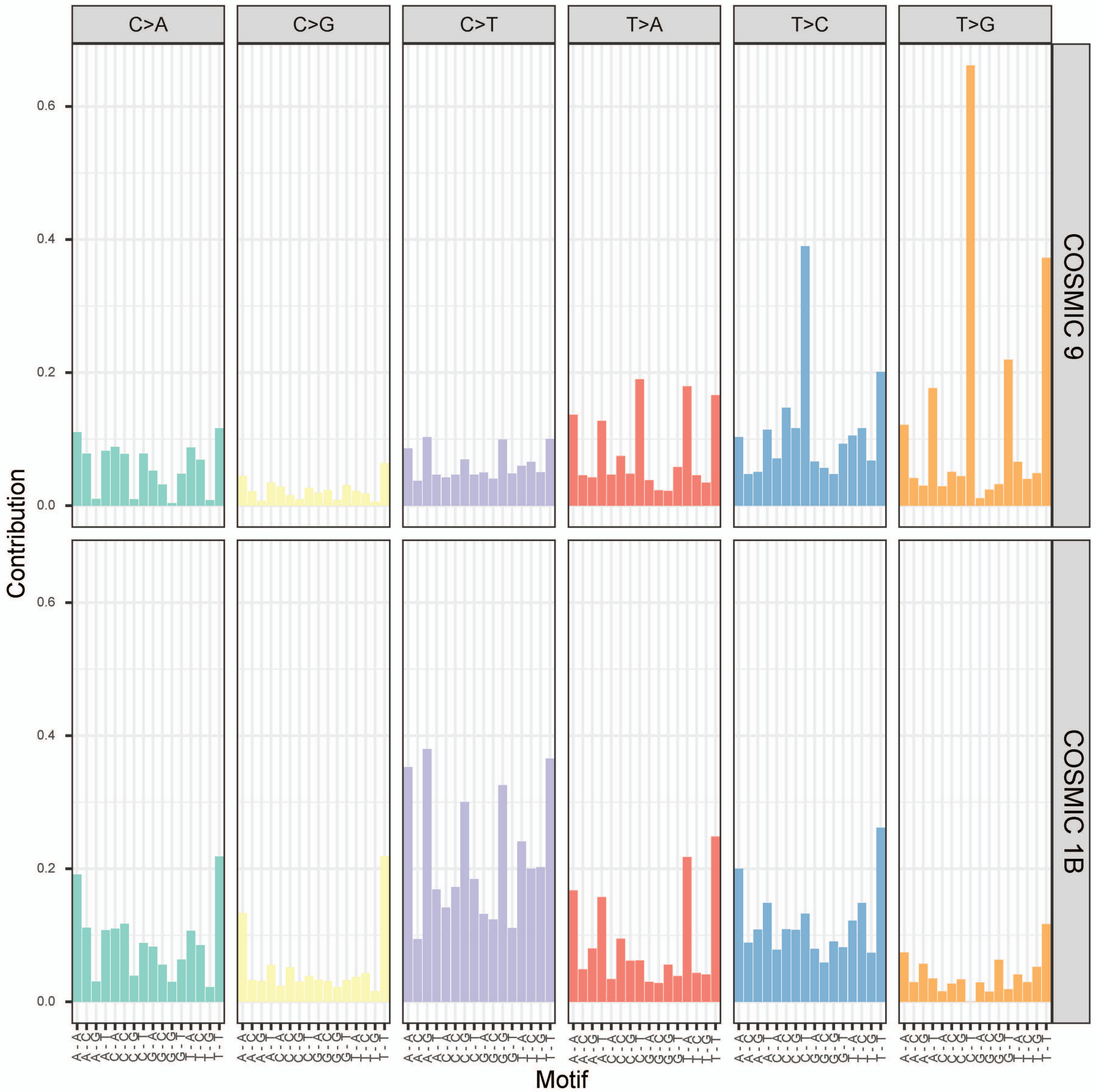

B.

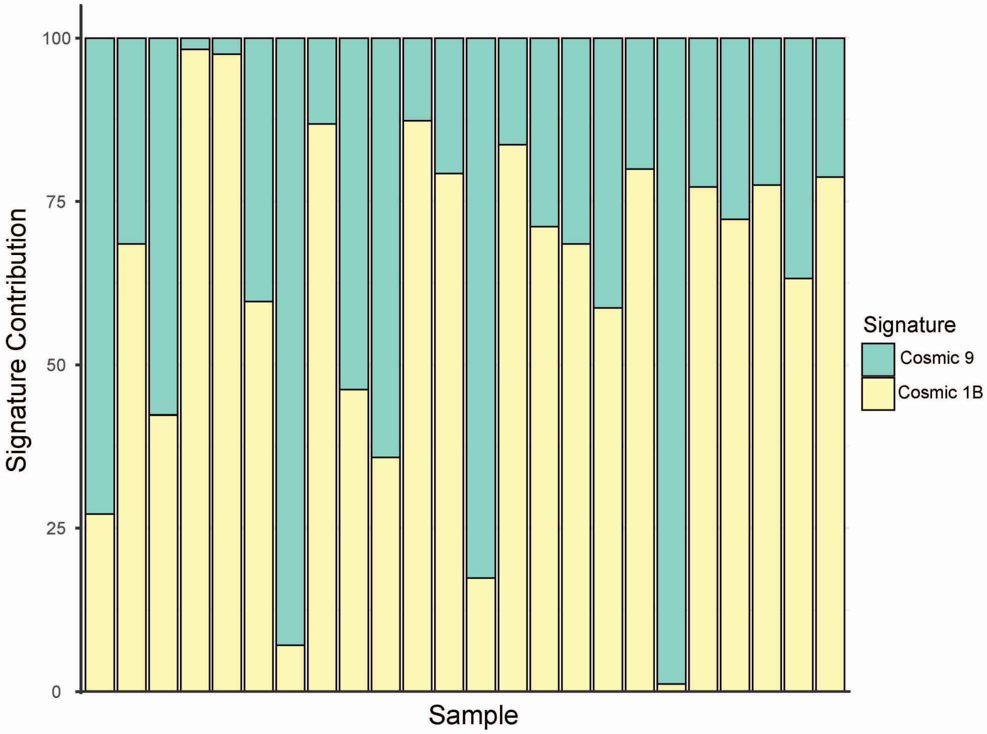

Supplemental Figure 2: Mutational signatures in WGS samples. (A) Two mutational signatures contributed to the variation in the n=24 WGS samples when considered alone. (B) Relative contribution of each mutational signature in WGS samples.

Supplemental Figure 3

A.

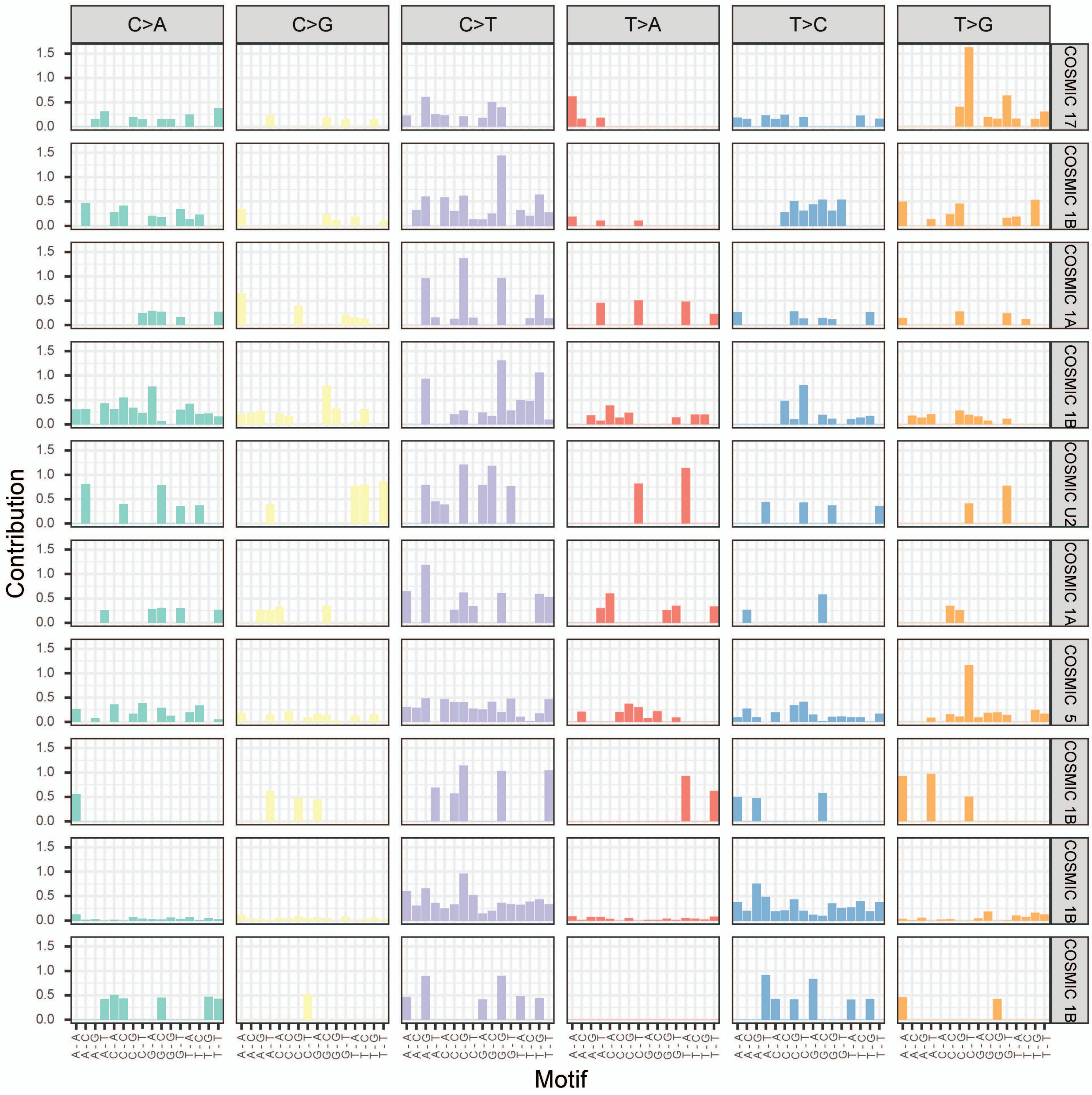

B.

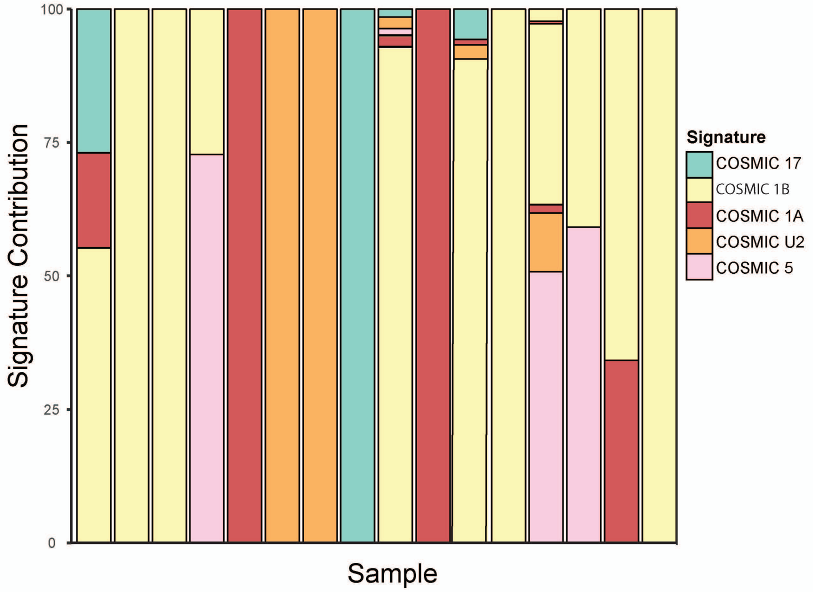

Supplemental Figure 3: Mutational signatures in WES samples. (A) Five mutational signatures contributed to the variation in the n=13 WES samples when considered alone. (B) Relative contribution of each mutational signature in WES samples.

Supplemental Figure 4

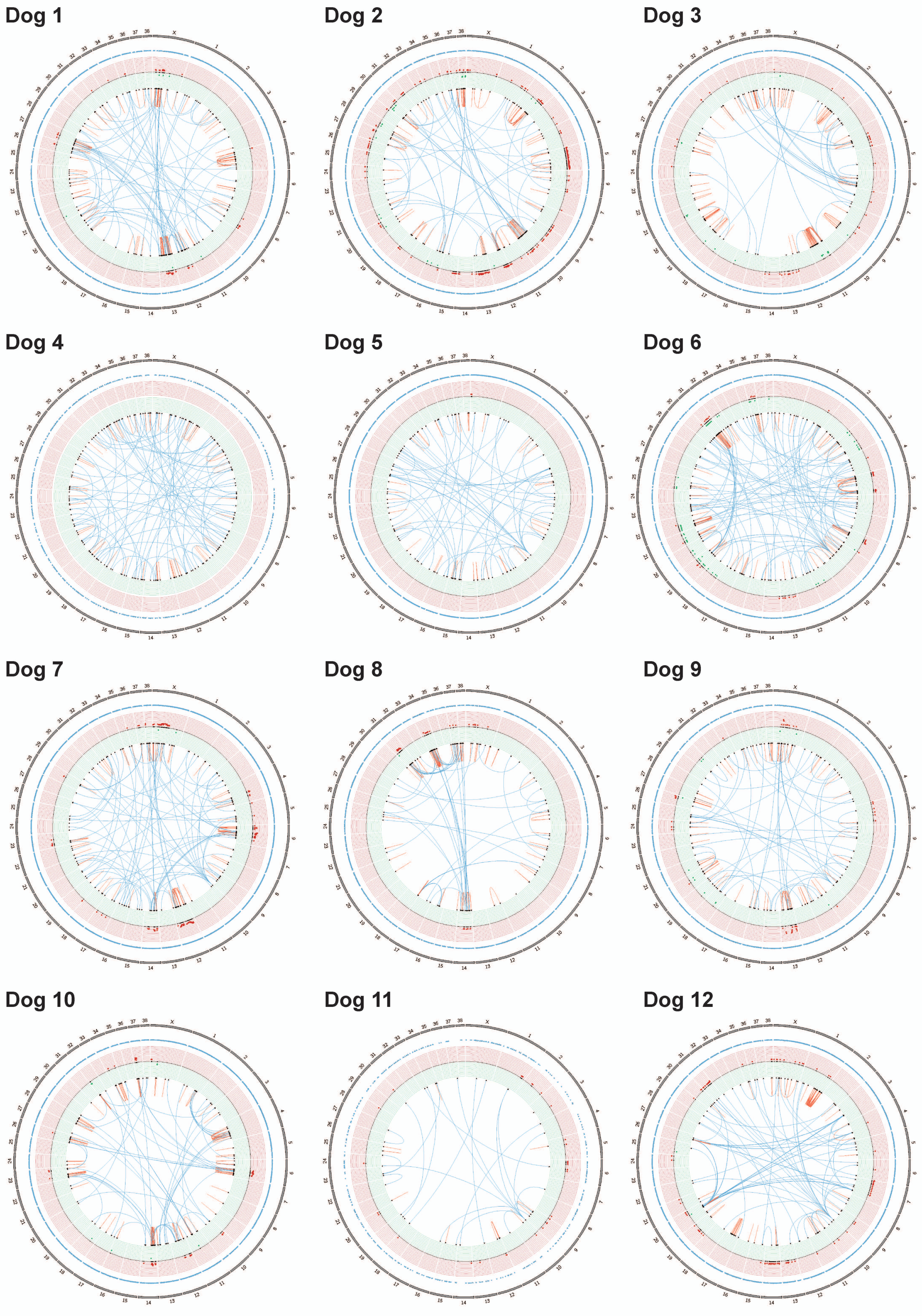

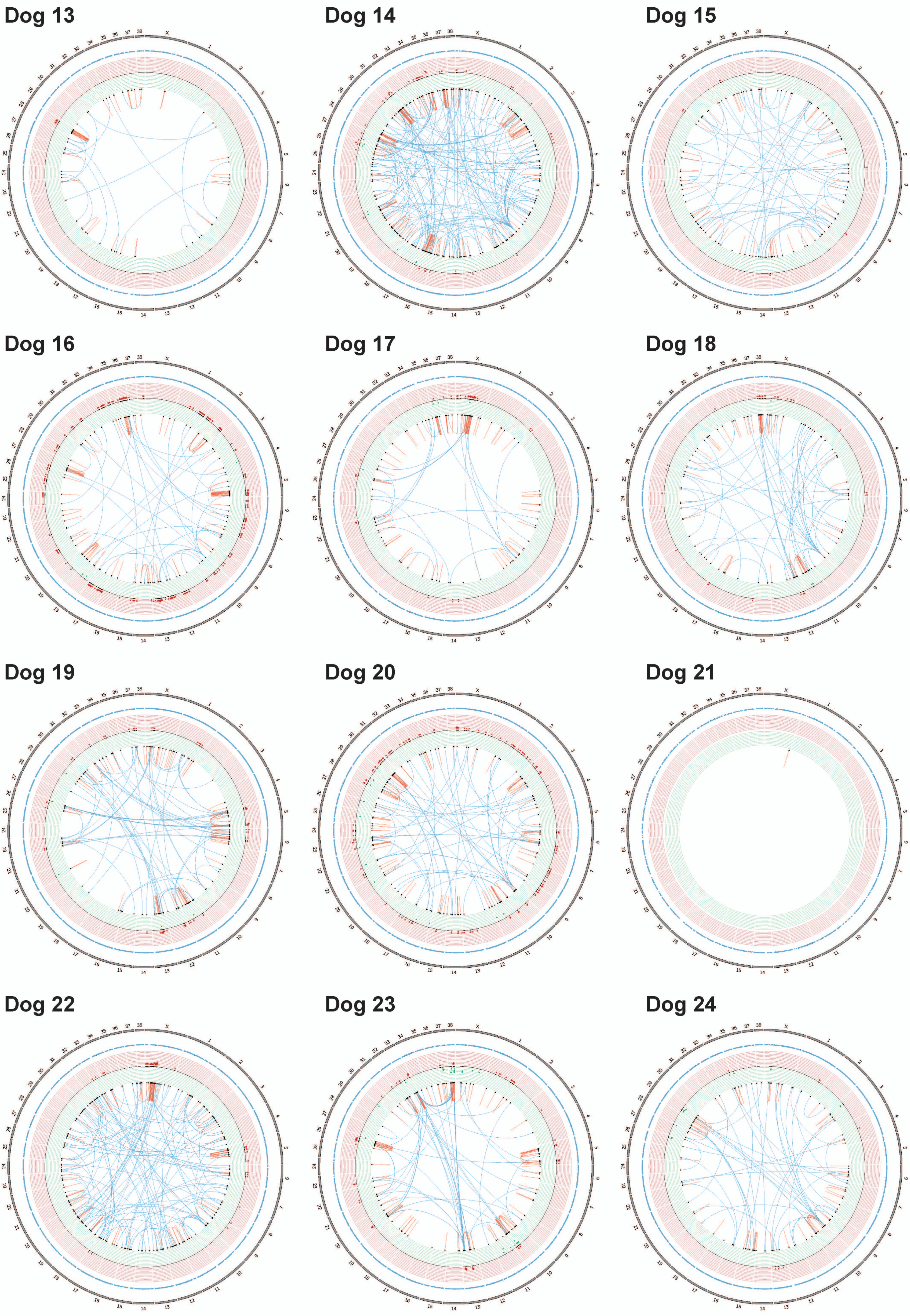
Dog 13
Dog 14
Dog 15
Dog 16
Dog 17
Dog 18
Dog 19
Dog 20
Dog 21
Dog 22
Dog 23
Dog 24

Supplemental Figure 4: Circos plots for WGS samples. Circos plots for each WGS sample. Blue triangles = SNVs; red dots = amplifications; green dots = deletions; dark red arrows = intra-chromosomal translocations; dark blue arrows = inter-chromosomal translocations.

Supplemental Figure 5

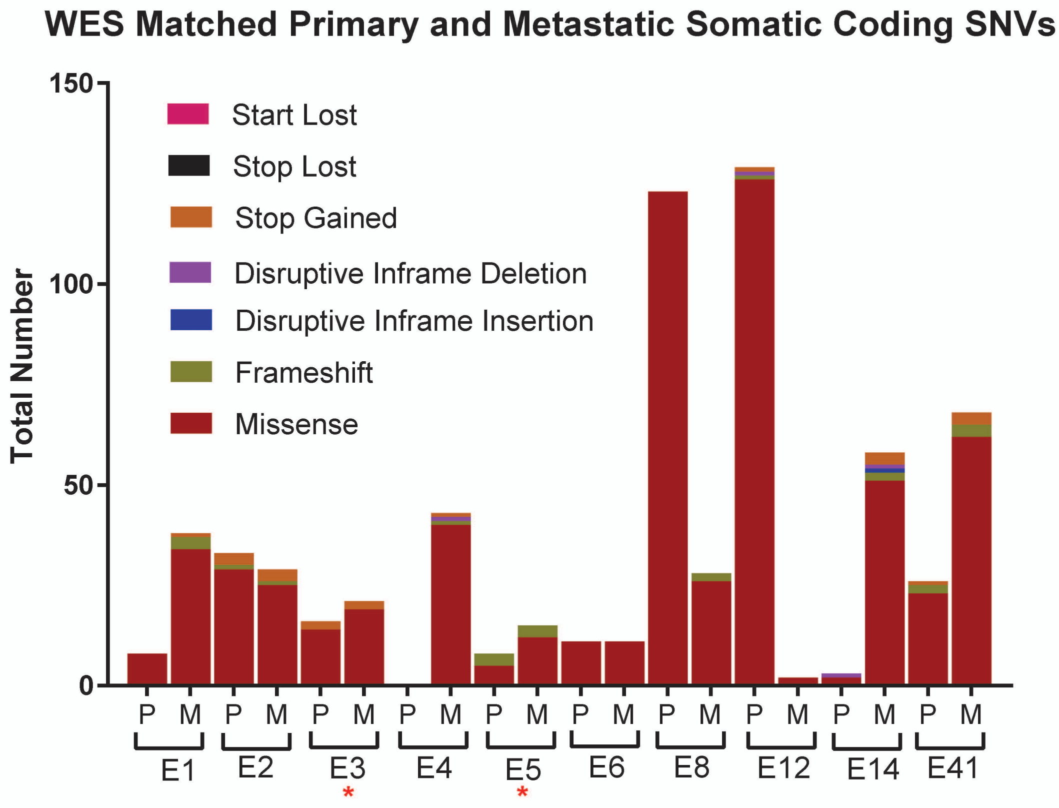

Supplemental Figure 5: Somatic SNVs Identified in Matched Primary and Metastatic OS. Bar chart illustrating frequency of somatic SNVs in matched primary and metastatic OS samples. Red (*) symbol = primary tumor biopsy was collected at the same time as the metastatic lesion at diagnosis. P = primary tumor. M = metastatic tumor.

Supplemental Fig 6

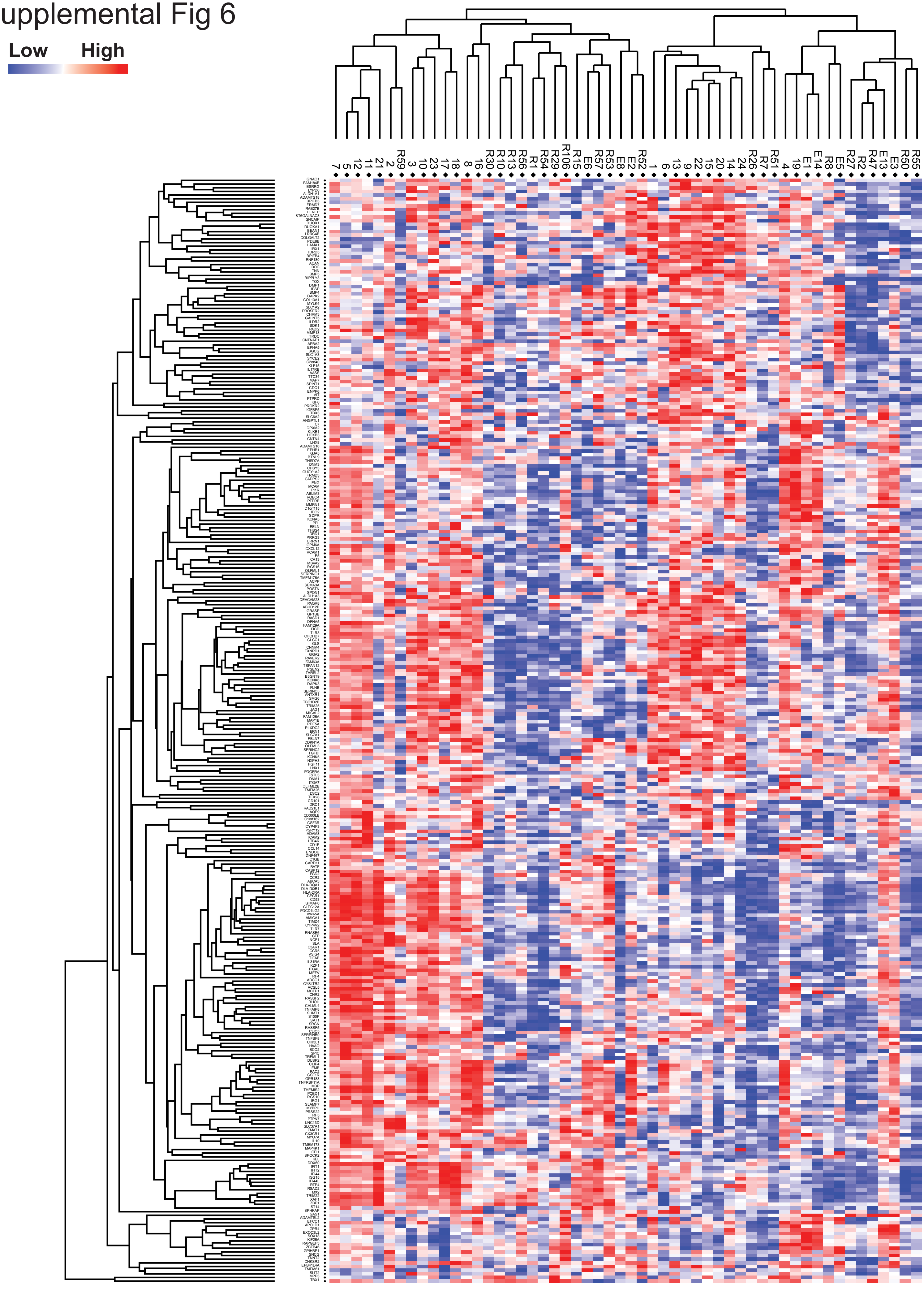

Supplemental Figure 6: Hierarchical clustering of primary canine OS. Hierarchical pairwise average-clustering was used with the similarity between gene expression (normalized HTSeq counts) values in n=54 primary OS samples for 302 differentially expressed genes measured by Spearman's rank correlation. In the heat map generated, Blue = decreased gene expression; Red = increased gene expression.
