# Supplementary material for: Canine osteosarcoma genome sequencing identifies recurrent mutations in DMD and the histone methyltransferase gene SETD2
Source: Commun Biol. 2019 Jul 19;2:266. doi: 10.1038/s42003-019-0487-2 (PMC6642146; doi:10.1038/s42003-019-0487-2)
Supplement: Supplementary file 2 — Description of additional supplementary items [file 42003_2019_487_MOESM2_ESM.docx]

**Titles and descriptions of supplementary data tables.**

**Supplementary Data 1: Sequencing Metrics.** Sequencing metrics are provided for the WGS, WES and RNA-seq samples.

**Supplementary Data 2: Analysis Tools.** All analytic tools, flag purposes and/or parameters used within the study.

**Supplementary Data 3: Cohort Demographics.** Age (years), sex, breed, primary tumor location and primary location of metastasis for WGS, WES and RNA-sequencing cohorts.

**Supplementary Data 4: Recurrently mutated and osteosarcoma-associated genes.** Genes with somatic aberrations associated with osteosarcoma and genes associated with germline predisposition for childhood cancer and/or canine osteosarcoma were used to filter variants.

**Supplementary Data 5: Germline Variants.** Germline variants in WGS and WES primary osteosarcoma samples.

**Supplementary Data 6: Somatic SNVs.** Somatic single nucleotide variants in WGS (primary) and WES (primary and metastatic) osteosarcoma samples. The human equivalent codon and COSMIC v87 incidence of somatic mutations at that site is provided for select cancer-associated genes.

**Supplementary Data 7: Somatic CNVs.** Somatic copy number variants and associated genes for WGS primary osteosarcoma samples, as determined using the tCoNut algorithm.

**Supplementary Data 8: Tumor Content Estimates.** Estimates of tumor content were determined for primary osteosarcoma samples based on hematoxylin and eosin sections of adjacent tumor sections.

**Supplementary Data 9:** **Extended Somatic CNVs.** Sub-homozygous and heterozygous somatic copy number variants across primary osteosarcoma WGS using the tCoNut algorithm.

**Supplementary Data 10: GISTIC Analysis.** GISTIC analysis of significant genomic regions recurrently impacted by somatic copy number changes based on amplitude, frequency, and chromosomal boundaries of these events.

**Supplementary Data 11: Somatic SVs.** Genes impacted by somatic structural variants across the primary osteosarcoma WGS samples.

**Supplementary Data 12: WES SNVs.** Candidate pathogenic somatic single nucleotide variants in matched primary and metastatic osteosarcoma samples evaluated via WES.

**Supplementary Data 13: HTSeq Counts for Differentially Expressed Genes.** HTSeq counts for n=54 primary osteosarcoma samples.

**Supplementary Data 14: Clade Comparison.** Comparison of differentially expressed genes when compared between clade 1 and clade 2. Genes that passed a p-adjusted value of =<1E-5 and a -2 <log2FC>2 were used as input for pathway analysis. The threshold for significance was set using a Benjamini-Hochberg p-adjusted value of 2.0E-6.

**Supplementary Data 15: Differentially Expressed Genes.** Thirty-one immune genes were differentially expressed between clade 1 and clade 2. The threshold for significance was set using a Benjamini-Hochberg p-adjusted value of 2.0E-6.
